# Mask dynamics in eye region–based person identification: Effects of mask removal and addition

**DOI:** 10.1167/jov.25.12.15

**Published:** 2025-10-07

**Authors:** Teresa Garcia-Marques, Manuel Oliveira, Paulo Ventura

**Affiliations:** 1 ISPA–William James Center of Research, Lisbon, Portugal; 2 Eindhoven University of Technology, Eindhoven, the Netherlands; 3 Faculty of Psychology, University of Lisbon, Lisbon, Portugal

**Keywords:** visual face identification, holistic processing, surgical mask

## Abstract

What is the impact of dynamic changes in facial visibility on identifying the eye and forehead region? This study examines how wearing or removing a mask affects the ability to visually identify the eyes. We investigate whether these changes impact the recognition of upper facial features and alter sensitivity to the misalignment of the face's upper and lower halves, which disrupts holistic face processing. Results show that removing a mask generally impairs visual identification, suggesting that the perception of the whole face hinders recognition of the upper half. This hindering is evident from the fact that the interference decreases when the face is misaligned. In contrast, the impairment in identification caused by adding a mask to a target face rises from losing original support of the holistic processing, given that it was not diminished when the upper and bottom halves were misaligned. Additional findings show that misalignment negatively affects the identification of faces where a mask was either maintained or added, suggesting that masks may actually help direct attention to relevant facial features, rather than being integrated into a holistic representation. We discuss these results in light of their theoretical and practical implications for visual identification, particularly in the context of dynamic changes to facial appearance.

## Introduction

Evidence indicates that face processing is typically holistic and that covering the lower half of the face with a surgical mask disrupts this perception, restricting face recognition (e.g., Freud et al., 2020; Garcia-Marques, Oliveira, & Nunes, 2022; Manley, Chan, & Wells, 2019; Roberson, Kikutani, Döge, Whitaker, & Majid, 2012). Evidence (Carragher & Hancock, 2020) shows that face-matching accuracy may drop by 34%–52% for masked faces compared to unmasked faces. This decline has occurred regardless of the observer's familiarity with the faces (e.g., Noyes & Hancock, 2021), highlighting the robust disruptive effect of masks on facial recognition. When the bottom half of the face is covered, it disrupts the perceptual integration of facial features, preventing individuals from accurately gauging the spatial relationships between original key facial features (Tanaka & Farah, 1993; Tanaka & Sengco, 1997).

However, it remains an empirical question how spatial relationships involving masked faces affect face recognition when we first meet a person wearing a mask. During encoding, the presence of a mask may establish spatial relationships that support face recognition. This suggests that, under these conditions, a mask could be integrated into the holistic processing of the entire visual percept rather than disrupting it. We will review evidence that challenges this idea before challenging it empirically.

### Masks and holistic processing

In various ecological contexts, researchers have examined the impact of different types of facial occlusions—such as ski masks, surgical masks, and other disguises (e.g., Hockley, Hemsworth, & Consoli, 1999; Mansour, Beaudry, & Lindsay, 2017; Mansour et al., 2020; Manley et al., 2019; Nguyen & Pezdek, 2017; Righi, Peissig, & Tarr, 2012; Stajduhare, Ganel, Avidan, Rosenbaum, & Freud, 2022)—on overall face recognition. In all these contexts, the removal of the initial facial occlusion presented during encoding has been shown to negatively affect face recognition and/or face-matching performance (Carragher & Hancock, 2020; Dhamecha et al., 2014). The introduction of a “new bottom half” of the face tends to disrupt positive identifications.

An experimental paradigm that helps to explain this phenomenon is the composite face task (e.g., Hole, 1994; Young, Hellawell, & Hay, 1987). In the composite face task, two parts of a face (e.g., the *upper and bottom halves*) are combined into a single composite image. Participants are typically asked to recognize or judge the identity of just one part of the face (e.g., the upper half). When the upper half of the face to be identified is aligned with a different bottom half, likely because we apprehend the stimuli holistically, it is difficult to focus on just one part without interference from the other. Importantly, misaligning the two halves of a face disrupts holistic processing, which can make the task easier by reducing interference from the irrelevant half. But for the same reason, misalignment is detrimental to recognition when the upper half is correctly paired with its corresponding lower half, since in this case, the unified whole face would be beneficial for recognition.

Righi et al. (2012) tested whether changes in the upper half of the face, such as hairstyle or the use of eyeglasses, interfere with performance on the composite face task. Their results showed that adding a disguise specifically to the test half of the face increased the perceptual weight given to the unmodified part. However, the reverse did not occur; the processing of the unmodified face part was not affected by the disguised half. This suggests that participants tend to focus on the uncovered half of the face and can effectively ignore the disguised half. In fact, some evidence shows that partially covering the face improves performance in recognizing emotions or detecting deception, possibly by blocking out irrelevant facial information (Kret & de Gelder, 2012; Leach et al., 2016).

The studies cited above agree that both the removal and addition of a mask to a face are detrimental to target recognition, which relies on holistic processing. The internal features hidden by a mask are crucial for configural processing and effective face encoding (e.g., Ellis, Shepherd, & Davies, 1979; Young, 1984; Young, Hay, McWeeny, Flude, & Ellis, 1985). When these regions are altered or hidden, it becomes more difficult to recognize a face as the same individual. However, when the task is one of recognizing the upper half of the face, Righi et al. (2012) suggest that by adding a mask to the bottom half and disrupting holistic processing, we may enhance the correct identification of the eye's region (see also Itier, Alain, Sedore, & McIntosh, 2007). This improvement would occur if masks isolated the features that need to receive our attention. If this is the case, then adding a mask to a previously known face would not only lead to better identification of the upper half but would also reduce the likelihood of benefiting from any misalignment between the two halves, which would aim to disrupt holistic processing (see Jin, Hayward, & Cheung, 2024).

This “mask as an isolated upper half” hypothesis is challenged in different ways. First, because we are more skilled in face processing (Richler, Wong, & Gauthier, 2011), which has a holistic nature, it is challenging to attend to specific parts of a face, as facial features are naturally integrated into a unified percept. Second, because most masks do not obscure the overall shape of the target face, which is somewhat preserved, it is likely that they allow this and other features of the percept to be integrated into the whole.

This “holistic mask integration” hypothesis is supported by the idea that top-down mechanisms can support the holistic perception of an incomplete percept (Zhao, Bülthoff, Bülthoff, 2016; see also Jiang, Blanz, & O'Toole, 2006; Russell, Biederman, Nederhouser, & Sinha, 2007). These top-down mechanisms likely enable perceptual features to be integrated into a single representation. Previous research has shown that facial parts are perceived as such because individuals tend to quickly integrate them into wholes. Even when a part is obscured, some form of perceptual inference occurs based on the size and shape of the occlusion itself. The key concept is that a mask may enhance the visual system's tendency to group contour elements when one contour continues smoothly from another, relying on top-down processes to “complete the good shape” (Wertheimer, 1923). If this is the case, the contours of the mask may be integrated into the overall percept, providing the same type of holistic access as an unmasked face, rather than isolating individual features. In this way, the masked face may form a “whole face,” representing an entity greater than the sum of its parts (e.g., McKone, 2004).

Within this alternative view, where masks are integrated into the whole percept, we would expect that when the task involves identifying only the upper half of a face, adding a mask to the original target would be similar to adding a “new bottom half” and that this type of interference could potentially be mitigated by misaligning the two halves of the face.

The removal of the mask from a studied target is expected to interfere with the recognition of the upper half of the face due to holistic processing. Therefore, misalignment is anticipated to generally improve performance in this encoding condition, independent of the alternative explanations discussed above.

### The current study

In our experiment, we address how holistic processing interferes with target identification through the eyes and forehead region when individuals are previously met with or without a mask. We approach features of holistic processing interference by contrasting identification performance when the upper and bottom halves of the face are presented aligned or misaligned (adapting this procedure from the composite face task to our goals). Better performance in misaligned conditions, which disrupt holistic processing, suggests that the bottom half is interfering with performance as a result of face holistic processing.

**Figure 1.**
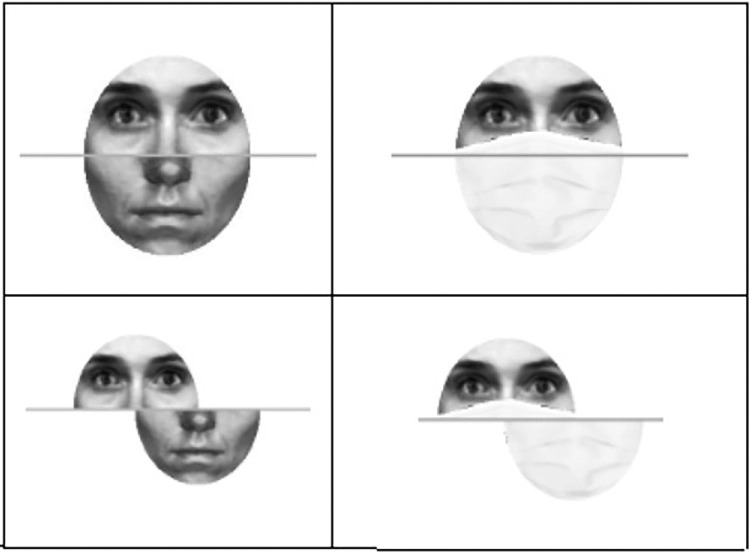
Illustration of the result of the masking procedure using facial landmark detection for alignment and misalignment versions.

We adapted the experimental procedures from the sequential matching paradigm (Farah, Wilson, Drain, & Tanaka, 1998). First, we familiarized participants with faces presented either with or without a mask. Participants were then asked to make recognition judgments based on the upper half of a face while ignoring the lower half. Each target had a version in which the lower half was presented either with or without a mask. In the relevant trials (positive identifications), the correct upper half was paired either with the corresponding bottom half (Mask–Mask, No Mask–No Mask) or with the opposite bottom half (Mask–No Mask, No Mask–Mask). Filler trials (negative identifications) involved other targets, with or without a mask, presented during the test phase (see Figure 1). In half of the trials, the target was shown with the upper and bottom halves aligned, while in the other half, they were misaligned.

As in Farah et al. (1998), the primary dependent measure used was the accuracy of recognition judgments. This measure allows for a direct test of our hypothesis by comparing performance across alignment and misalignment conditions under different encoding contexts. Within our experimental paradigm, and congruent with previous studies, we conceptualize holistic processing as influencing overall performance (i.e., the proportion of correct responses), rather than expecting our manipulation to selectively affect either correct identifications (hits) or incorrect ones (false alarms).^1^

## Method

### Participants and design

Participants were recruited from multiple English-speaking countries via Prolific Academic. We targeted the identification of a moderate effect size in main effects and interaction in a repeated-measures analysis of variance (ANOVA) associated with our design, having α = 0.05 and power = 0.90. The software G-Power suggested a sample size of 30 participants. Our 30 participants were 50% females and 50% males with *M*_age_ = 27.8 years (*SD* = 6.32).

Trials performed defined the within-participant design, characterized by 2 (study phase: face with vs. without mask) × 2 (test phase: face with vs. without mask) × 2 (aligned vs. misaligned).

### Material and task

The stimuli were the same as those used by Ventura et al. (2013). Female and male faces from the MPI face database (Troje & Bülthoff, 1996), converted to grayscale, were cut in half, and different composites were made of the upper and bottom halves of different faces. A line separated face halves, and faces were presented inside an oval within a black rectangle.

Facial masking was implemented using the OpenCV v3.4.2 and dlib v19.19 modules in a Python 3.7 environment. A masked version of each face image was created by overlaying a medical-looking facial mask onto each face image. Each mask image was automatically resized and adjusted to the facial shape of each face stimulus (aligned version), using facial detection and landmarking algorithms (dlib module). A misaligned version of each stimulus was then created by extrapolation from the masked aligned versions (Figure 1).

**Figure 2.**
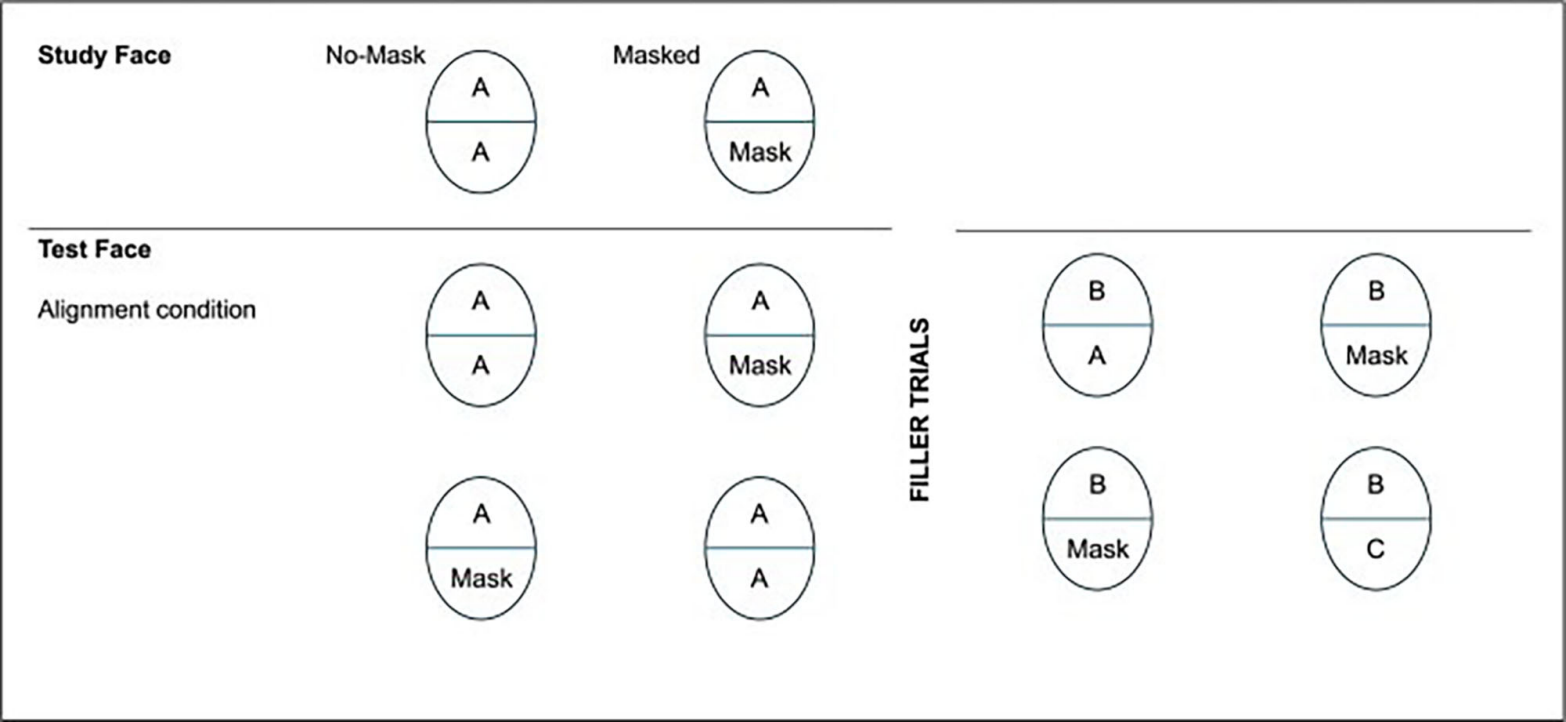
Alignment materials that support experimental conditions. The misalignment material replicates this pattern.

For the sequential matching task (of upper halves), we created the eight types of trials defined by the conditions of the 2 (study phase: face with vs. without mask) × 2 (test phase: face with vs. without mask) × 2 (aligned vs. misaligned) design; see Figure 2. Two sets of stimuli allowed participants to see one photo only once in one experimental condition. Each set comprised 368 trials (24 trials for each condition).

### Procedure

Data were collected using Qualtrics software. Participants were invited to participate in a face perception study with a duration of 7 to 10 minutes. After providing informed consent, each participant was randomly assigned to one of two counterbalanced material conditions (368 trials in four blocks). The general instructions emphasized that participants’ task was to recognize a target face, which could be shown either with or without a surgical mask. Immediately afterward, they were presented with a second image—also either masked or unmasked—and were asked to judge whether the upper half of the face belonged or not to the previously seen target. It was also emphasized that in half of the trials, the upper and bottom halves of the face were spatially separated during the identification phase.

Each trial began with a blank screen for 1 second, followed by the study stimulus (the target composite face) presented at the center of the screen for 0.8 seconds. After an interstimulus interval of 0.5 seconds, the test stimulus appeared for 0.5 seconds. The judgment stimulus could consist of either the same upper half (masked or unmasked) presented during the study phase or a different one (filler trials), also either masked or unmasked. These were presented in either aligned or misaligned configurations, with all conditions randomly intermixed. Participants were instructed to focus on the upper half of each face and to judge whether it was identical to the one they had just seen.

## Results

Participants demonstrated high compliance with task instructions, with accuracy ranging from 87% to 99% across conditions. We expect the rate of correct responses (CRs) to be influenced by all our experimental manipulations, potentially resulting in a three-way interaction between the conditions. Specifically, by adding a mask to a face, we create the kind of disruption that is expected to be reversed when the mask is removed. Statistical analyses were conducted in *Jamovi*,^2^ using a repeated-measures ANOVA applied to all design factors, with CR rate and hits (correct identifications) as dependent measures (see Table 1).

**Table 1.** Repeated-measures ANOVA results for correct responses and identifications.

	Correct response rate	Hits (correct identifications)
Study effect	*F*(1, 29) = 10.93, *p* = 0.003, $\eta_{p}^{2}$ = 0.27	*F*(1, 29) = 4.10, *p* = 0.052, $\eta_{p}^{2}$ = 0.12
Test effect	*F*(1, 29) = 4.84, *p* = 0.036, $\eta_{p}^{2}$ = 0.14	*F*(1, 29) = 5.73, *p* = 0.023, $\eta_{p}^{2}$ = 0.17
Alignment	*F*(1, 29) = 2.55, *p* = 0.121, $\eta_{p}^{2}$ = 0.08	*F*(1, 29) = 1.28, *p* = 0.*268*, $\eta_{p}^{2}$ = 0.04
Study × Test	*F*(1, 29) = 53.70, *p <* 0.001, $\eta_{p}^{2}$ = 0.65	*F*(1, 29) = 76.87, *p <* 0.001, $\eta_{p}^{2}$ = 0.73
Study × Alignment	*F*(1, 29) = 1.52, *p =* 0.228, $\eta_{p}^{2}$ = 0.05	*F*(1, 29) = 7.78, *p* = 0.009, $\eta_{p}^{2}$ = 0.21
Test × Alignment	*F*(1, 29) = 7.11, *p* = 0.012, $\eta_{p}^{2}$ = 0.20	*F*(1, 29) = 26.15, *p <* 0.001, $\eta_{p}^{2}$ = 0.47
Study × Test × Alignment	*F*(1, 29) = 6.05, *p* = 0.020, $\eta_{p}^{2}$ = 0.17	*F*(1, 29) = 15.66, *p <* 0.001, $\eta_{p}^{2}$ = 0.35

### Correct responses

As expected, study and test conditions interacted, with matching generally improving performance. Being significant in both conditions, the benefit of matching is stronger for faces tested with a mask (Matched: *M* *=* 0.92, *SE* *= *0.02; Mismatched: *M = * 0.88, *SE =* 0.02, *t*(29) = 7.98; *p <* 0.001, *d* = 1.46), compared with faces tested without a mask (Matched: *M =* 0.84, *SE =* 0.02; Mismatched: *M =* 0.88, *SE =* 0.02; *t*(29) = 4.04; *p <* 0.001, *d* = 0.74).

**Figure 3.**
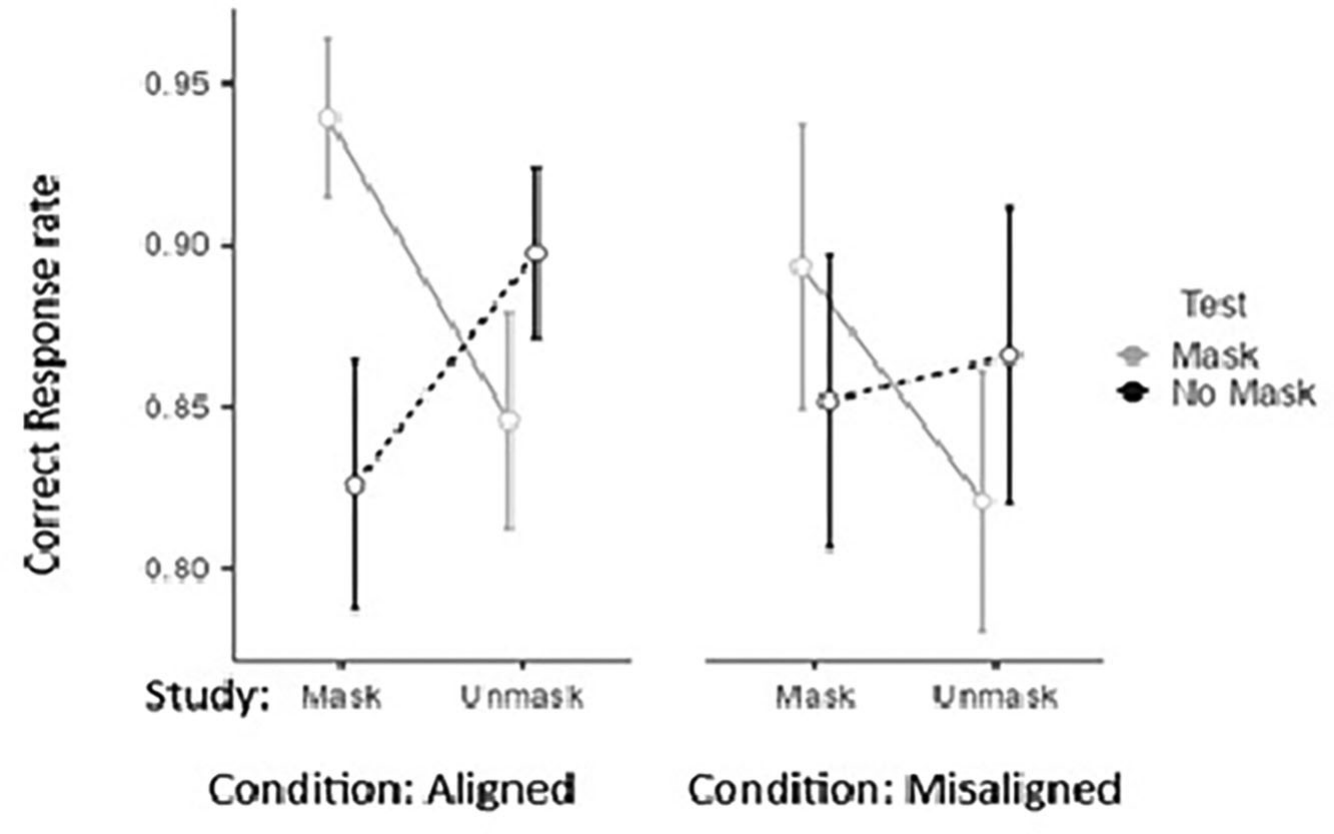
Proportion of correct responses for faces studied with and without a mask, when tested in the same conditions or in reverse conditions, under both aligned and misaligned conditions.

Although the Study × Test interaction pattern is significant in both alignment, *F*(1, 29) = 42.15, *p <* 0.001, $\eta_{p}^{2}$ = 0.60, and misalignment, *F*(1, 29) = 16.67, *p <* 0.001, $\eta_{p}^{2}$ = 0.36, conditions, alignment moderates the effect (see Table 1 and Figure 3). This occurs because misalignment interferes with the matching benefit of unmask, suggesting that the matching advantage in the alignment context rises from holistic processing. It does not interfere with the matching effect occurring for faces tested with a mask, suggesting that the benefits of matching and the interference of mismatching are not promoted by holistic processing.

Overall, recognition performance was better for tests of masked targets (*M* *=* 0.88, *SE* *=* 0.02) than for tests of unmasked ones (*M* *=* 0.86, *SE* *=* 0.02), suggesting that masks were simplifying the task of focusing on the eye region of the target. Also, faces studied with a mask (*M* *=* 0.88, *SE* *=* 0.02) led to general better performance than those studied without one (*M* *=* 0.86, *SE* *=* 0.02). The Test × Alignment interaction also clarifies that having the test with a masked face (matched or unmatched to study condition) is generally more favorable for the alignment conditions (*M*_Mask_ = 0.89 vs. *M*_Unmask_ = 0.86) than for the misalignment condition (*M*_Mask_ = 0.86; *M*_Unmask_ = 0.86). This general negative effect of misalignment suggests that masks may actually help direct attention to relevant facial features, rather than being integrated into a holistic representation.

### Hits^3^

When we isolated only the rates of correct positive identifications (hits) in the analysis, the results matched those previously isolated with the CR rates. Here we just focus the presence of the Study × Test interaction representing the matching effect for unmasked (match 0.93 vs. mismatch 0.75) and masked photos (match 0.92 vs mismatch 0.80). The target three-way interaction reported the same effects observed with CRs; a cross-interaction representing a clear matching effect for alignment conditions, *F*(1, 28) = 61.21, *p <* 0.001, $\eta_{p}^{2}$ = 0.70, was not found in the misalignment conditions. Although the interaction was there, *F*(1, 28) = 151.80, *p <* 0.001, $\eta_{p}^{2}$ = 0.64, there was no impact of matching when the test was made with the whole face when the two parts were misaligned, *t*(29) =1.58, *p* = 0.403, *d* = 0.29.

Knowing that both study and test conditions are relevant for our ability to perceive a person through their eyes, we approach a more direct test of the level of the interference promoted by adding a mask compared with the interference of taking out the mask. For this, we run a simple effects analysis over CR. Isolating the focused interaction by study conditions, data show the following:

*When we know the person without a mask,* the addition of the mask disrupts the identification of the upper half, *F*(29) = 22.22, *p <* 0.001, $\eta_{p}^{2}$ = 0.43, and misalignment has no relevant role (no interaction component), *F*(29) = 0.14, *p* = 0.714, $\eta_{p}^{2}$ = 0.00. Adding a mask, much like misalignment, appears to disrupt holistic face processing.
*When we meet a person with a mask*, and the mask is taken off, this disrupts the identification of the person through the eyes (upper half) compared with the matched mask target condition, *F*(1, 29) = 46.14, *p <* 0.001, $\eta_{p}^{2}$ = 0.61. Documenting the interference of the holistic process, the effect is qualified by the alignment conditions, *F*(29) = 10.71, *p* = 0.003, $\eta_{p}^{2}$ = 0.27. When holistic processing is disrupted by misalignment, mismatch interference is significantly reduced, leading to improved performance. However, importantly, the mask study/test match advantage is diminished when misalignment occurs, suggesting that the mask can drive attention to the “undisguised half” (as suggested by Righi et al., 2012).

## Discussion

The results of the experiment show that encountering a person with a mask and then seeing them without it (a mismatch condition) impairs the identification of the upper half of the face that was initially isolated by the mask. This outcome is expected, as removing the mask allows for holistic processing of the full face, which can interfere with the perception of the previously isolated features. This interference is further supported by improved performance in the misaligned condition, suggesting a release from holistic interference (e.g., Richler et al., 2014). Our results align with the idea that misalignment, by disrupting the ability to process spatial relationships between facial features, generally impairs recognition of the upper half—except in cases like this, where it reduces interference from the irrelevant bottom half.

The results also show that adding a mask during the test phase—a mismatch condition—negatively affects the identification of a person's eyes. This mirrors previous findings of a general detrimental effect of masks on face recognition (e.g., Garcia-Marques et al., 2022; Freud et al., 2020; Manley et al., 2019; Roberson et al., 2012). One likely reason for this impairment is that the mask obstructs the ability to accurately perceive the spatial relationships between key facial features, as suggested by earlier studies (Tanaka & Farah, 1993; Tanaka & Sengco, 1997). Supporting this interpretation, misalignment does not improve performance in this condition—further indicating that the mask disrupts configural processing rather than simply introducing bottom-half interference.

A third important finding from our experiment is that when it comes to identifying the upper half of a face, wearing a mask appears to guide attention more effectively to those features—an effect even stronger than that produced by misalignment. When the bottom half of the face is misaligned, identification of masked targets is impaired. If this effect had occurred only for faces that were previously studied with a mask, one might argue that the mask was integrated into a holistic representation and that misalignment disrupted this perceptual whole. However, the same pattern of results was observed even when faces had originally been studied without a mask. Therefore, the more parsimonious explanation is that adding a mask to the bottom half of the face enhances recognition of the eye region by isolating the features that require focused attention (Itier et al., 2007; Righi et al., 2012). These results match the eye-tracking study data from Hsiao, Liao, and Tso (2022), who showed that participants who focused on the eye region during the recognition of masked faces performed better, suggesting that directing attention to the visible features, such as the eyes, can mitigate the challenges posed by mask occlusion.

In sum, our results offer no support for the “holistic mask integration hypothesis.” When perceiving a face flattened at the bottom by a mask, it is unlikely that the mask is integrated as part of the whole face. Instead, our findings support the “mask as an isolator of the upper half” hypothesis, indicating that the upper half of a masked face is processed independently of the lower half. Nevertheless, it is important to acknowledge that the use of computer-edited masked images likely fails to fully capture the range of perceptual information conveyed by naturally worn masks. Unmasked facial features can lead to subtle mask deformations, which may better integrate the mask into holistic face processing. This issue has important ecological and theoretical implications and should be explored in future research.

The matching study and test effect can be related to several memory phenomena, such as context-dependent memory (e.g., Eich, 1980; Godden & Baddeley, 1975), transfer-appropriate processing (e.g., Morris, Bransford, & Franks, 1977), and encoding specificity (Tulving, 1972). In addition to these likely determinants of performance, our study shows that for unmasked faces, participants rely on holistic perception to help identify specific features of the upper half of the face (the “whole-face advantage”; Tanaka & Sengco, 1997). However, the same mechanism does not explain the matching advantage for masked faces, as our results indicate that the mask is not integrated into the overall percept. Encoding specificity and the transfer of the same processes provide a better explanation for this advantage, as participants isolated the relevant part of the face for proper identification during the study and test phases.

We believe that our findings are highly relevant for understanding social interactions, particularly in applied contexts such as health care settings (see Lee, Cormier, & Sharma, 2022). The data suggest that individuals may experience greater difficulty identifying a health care worker after they remove their mask than when they put one on. These insights may also inform practices related to eyewitness testimony, which plays a crucial role in many criminal investigations (e.g., Or, Lim, Chen, & Lee, 2023), especially in situations where individuals partially conceal their identity and only the eye region remains visible. Our results align with findings by Manley et al. (2019), emphasizing that matching the test face to the original encoding condition is critical—presenting the full face can, in some cases, impair identification. Even when the encoding phase does not involve a mask, it may be beneficial to present a masked face during the identification phase if the eye region is considered most diagnostic.

### Limitations and future research

Given the specific aim of our study, the design does not allow us to draw firm conclusions about the underlying processes that contribute to improved identification of the upper half of a target face. Previous research has documented a mask bias effect, in which individuals are more likely to accept a masked face as one they have previously seen compared to an unmasked face (Garcia-Marques et al., 2022; Guerra et al., 2022; Or et al., 2023). Future studies could address this limitation by including appropriate filler trials and control conditions, in which the same upper half is paired with different bottom halves—as is standard in the full composite face paradigm. This would enable the application of a signal detection approach, providing a more nuanced assessment of the underlying recognition processes.

It is also important to note that the immediate identification context used in our study may not readily generalize to recognition settings, where masks have consistently been shown to impair performance (Guerra et al., 2022; Hsiao et al., 2022; Or et al., 2023). Furthermore, with respect to full face recognition, previous research has shown that the poorest performance tends to occur when both the study and test phases involve masked faces (Garcia-Marques et al., 2022)—a pattern that was not replicated in the present study. Future research should explore how immediate, memory-based identification processes extend to longer-term memory in similar contexts.

## Conclusions

Our main conclusion is that a perceptual feature not naturally part of the human face—such as a mask—is unlikely to be integrated into the holistic perceptual representation stored in memory. The results also show that masks help direct attention to the eye and forehead region when that is the individual's processing goal. Furthermore, putting on a mask interferes less with the immediate identification of a person via their eye region than removing a previously present mask. When the mask is removed, it disrupts the perception of the upper facial region (eyes and forehead), and participants may feel compelled to process the lower half of the face to gain confidence in recognizing the person as someone they had previously seen wearing a mask.

## Footnotes

1 As discussed by Gauthier and Bukach (2007; see also Richler, Floyd, & Gauthier, 2014), our design uses a partial version of the composite face task. In this setup, applying signal detection theory is problematic due to the lack of fully matched filler trials across all conditions, which limits the reliable estimation of false alarms. Participants typically view faces in which only the upper half is relevant, while the bottom half is either irrelevant or mismatched. As a result, the bottom half is less likely to produce incorrect identifications directly; however, it may still influence perception, complicating the interpretation of response biases.

2 Raw data and analysis can be found at https://osf.io/82pkx/?view_only=0adbbf1bc837453eac2cb93642594bc1.

3 We do not report false alarm results, given their unreliable status and the fact that their analysis does not encompass the study conditions, since the stimuli analyses are all “new” (so not studied). Nevertheless, we inform the reader that only the main effect of test is significant for this dependent measure, *F*(1, 29) = 5.73, *p <* 0.001, $\eta_{p}^{2}$ = 0.41, because faces tested without a mask were more prone to false alarms than the ones studied with the mask (*M* *= *0.86 vs. *M* *= *0.83). In addition, we clarify that all the effects observed in the hits analysis were replicated with an unreliable *d*′, and the analysis of an unreliable *c* suggests a bias component contributing to all those significant effects.
